# Product mix and time since cessation among Korean former smokers using non-combusted nicotine products: a KNHANES analysis with implications for lung cancer risk comparisons

**DOI:** 10.64898/2026.08.11.26360188

**Published:** 2026-08-13

**Authors:** S Cook, G Cohen, KM Cummings

**Affiliations:** 1 University of Michigan, Ann Arbor, MI; 2 Rose Research Center, Raleigh, NC; 3 Medical University of South Carolina, Charleston, SC

## Abstract

**Background.:**

Observational comparisons of former smokers who use non-combusted nicotine products with former smokers who quit without them require that two quantities be measured precisely: which product is being used, and how long ago cigarette smoking stopped. Neither quantity is recorded by the National Health Insurance Service (NHIS) screening instrument used in a recent Korean cohort study of post-cessation e-cigarette use and lung cancer risk. We characterized both quantities in a contemporaneous, nationally representative survey of the same population.

**Methods.:**

We analyzed the public-release microdata of the Korea National Health and Nutrition Examination Survey (KNHANES), 2018 to 2023, restricted to adults aged 19 years and older. Former smokers were identified by smoking status, and cessation duration was taken from the item recording months since the last cigarette. Former smokers currently using a heated tobacco product (HTP) or an e-cigarette (EC) were compared with former smokers using neither. KNHANES 2018 asked a generic e-cigarette question and, separately, a checklist naming HTP brands, allowing the two product classes to be separated. Distributions were compared with rank-based methods, the age–duration relationship with Theil–Sen regression, and residual imbalance by restricting the comparison group to respondents age-matched to within two years.

**Results.:**

The 2018 analytic sample comprised 1,348 former smokers, of whom 43 currently used HTP or EC and 1,305 used neither. Among the product-using former smokers, 58% reported HTP use without e-cigarette use, 21% reported both, and 21% reported e-cigarette use without HTP use; 79% reported any HTP use. Median cessation duration was 0.7 years (IQR 0.25 to 1.5) among product users and 12.0 years (IQR 5.0 to 20.0) among those using neither (Kolmogorov–Smirnov D = 0.76, P < 0.001), with the product user having quit more recently in 92% of cross-group pairs. The separation persisted within the short-term (<5 year) stratum (D = 0.34, P < 0.001; 73% of pairs) and after age matching, where the residual gap was 9.3 years. Cessation duration rose with age among those using no product (Theil–Sen slope +0.30 years per year) but was flat among product users (−0.01). Restricting to the screening-eligible stratum used in the cohort’s high-risk analysis did not attenuate the imbalance: among those aged 50 to 80, median cessation among no-product quitters rose to 15.5 years (n = 858), and adding a 20 pack-year criterion left 421 no-product quitters with a median of 11.0 years against three HTP/EC users who had quit 0.25, 1.0 and 2.0 years earlier, despite closely matched cumulative exposure (mean 37.6 vs 37.7 pack-years). The overall contrast reproduced in every wave from 2018 to 2023, with an age-matched residual of 9 to 11 years.

**Conclusions.:**

In a nationally representative survey of the same population and the same calendar year as the NHIS screening cohort analyzed by Kim et al., Korean former smokers using non-combusted nicotine products differed from other former smokers in two respects that bear directly on how such comparisons should be read. First, they were predominantly HTP users: 79% reported any HTP use, and only 21% reported e-cigarette use without HTP use. Second, they had stopped smoking approximately a decade more recently, a difference that survived stratification at five years and exact age matching. Neither quantity is recorded in the NHIS screening instrument. Cohort estimates comparing post-cessation product users with other quitters should therefore be interpreted with caution if they do not precisely characterize product composition and to time since cessation, and future studies should measure both directly.

## Introduction

Cigarette smoking is the dominant cause of lung cancer, and risk after cessation declines gradually over years to decades rather than abruptly.^1,2^ Any observational comparison between former smokers who adopt a non-combusted nicotine product and former smokers who quit without one is therefore, in substantial part, a comparison of cigarette exposure histories. Two quantities govern whether such a comparison is interpretable: the identity of the product actually used, and the time elapsed since the last cigarette.

Both quantities are difficult to capture in administrative cohorts. Kim et al. recently reported, in a National Health Insurance Service (NHIS) screening cohort of approximately 4.5 million Korean adults, that former smokers who used e-cigarettes after cessation had a higher lung cancer incidence than former smokers who quit without them; an accompanying commentary interpreted the result as evidence about the long-term health risks of e-cigarettes.^3,4^ The study has clear strengths, including its scale and its use of a switcher-versus-quitter comparison consistent with recommended practice for non-randomized studies of tobacco product exposure.^5^ Its exposure instrument, however, was a single question about “e-cigarette” use in the previous month, and cessation timing was inferred from smoking status recorded at two screening waves five years apart. The instrument therefore neither identifies which non-combusted product a respondent used nor dates the cessation event.

These limitations matter particularly in Korea in 2018, the baseline year of that cohort. Heated tobacco products (HTPs), including IQOS and lil, entered the Korean market in mid-2017 and were in widespread use by 2018.^6–8^ Where a survey instrument asks about e-cigarettes without naming HTPs, the resulting exposure group may be composed substantially of HTP users. A published KNHANES analysis has independently cautioned that some 2018 respondents using cigarettes and HTPs may have been classified as cigarette-and-e-cigarette users.^9^ Separately, a commentary by Shields raised three concerns about the Kim et al. analysis: that six years of follow-up may be too short to separate a causal effect from reverse causation; that exposure, including continuity of product use, is poorly characterized and HTP use unverified; and that the exposed cells are small relative to the effect sizes claimed.^10^ Reverse causation cannot be quantified from published data, but product composition and cessation timing can be measured directly in an independent source.

The Korea National Health and Nutrition Examination Survey (KNHANES) is the national reference source for tobacco use surveillance in Korea. It is nationally representative, fielded annually, and draws on the same adult population and the same calendar years as the NHIS screening cohort.^11,12^ In 2018 it recorded both quantities that the NHIS screening item does not: it asked separately about e-cigarettes and about HTPs by brand name, and it recorded time since the last cigarette in months.

We therefore used KNHANES to characterize, in the Korean population at the relevant time, the product mix and the cessation-duration distribution of former smokers using non-combusted nicotine products, relative to former smokers using none. This is a descriptive survey analysis and not a replication: KNHANES contains no lung cancer outcomes and cannot reproduce, correct, or refute any hazard ratio. Its purpose is to establish what the exposure group in a cohort of this design is likely to consist of, and how far apart the compared groups are on a variable that the cohort could not measure.

## Methods

### Data source.

All analyses use the public-release microdata of the Korea National Health and Nutrition Examination Survey (KNHANES) from 2018 to 2023.^12^ KNHANES is a nationally representative, annually fielded cross-sectional survey conducted by the Korea Disease Control and Prevention Agency.^11^ Kim et al. neither analyze nor cite KNHANES; we use it because it is the national reference source for tobacco use surveillance in Korea, and because in the 2018 baseline year it records the two quantities the NHIS screening item does not.

### Product items.

The KNHANES 2018 questionnaire included a first question asking about current e-cigarette use (questionnaire item Q11-1; variable BS12_2). Here the term e-cigarette was generic and not specified to be “liquid nicotine-containing.” It was then followed by a checklist (questionnaire item Q12-1; heated tobacco option, variable BS12_47) asking whether certain products were currently used, excluding e-cigarettes, one of the choices being heated tobacco products, defined with specific examples (IQOS, glo). A respondent answering affirmatively to question 12 but negatively to question 11 was therefore exclusively using HTP; yes to 11 and no to 12 corresponds to exclusive liquid EC use; yes to both is ambiguous and could mean either dual use of both or exclusive HTP use. See Figure S1 for the questions as asked in Korean. From KNHANES 2019 onward the questions are more specific: question 4-1 asks about current HTP use with examples, followed by question 5-1 on liquid nicotine EC use (Figure S2). From 2019 the HTP question is standardized into daily, occasional and former categories with frequency also asked, refining the product distinction it already drew in 2018 rather than introducing one.

### Population and definitions.

Analysis is restricted to adults aged 19 years and older. The smoking-status item is administered only to respondents who have smoked, so BS3_1 = 3 identifies former smokers without a further lifetime-use filter. Current smokers are BS3_1 in {1 (daily), 2 (occasional)}; former smokers are BS3_1 = 3. Cessation duration is BS6_4, recorded in months and divided by 12, excluding codes of 8888 and above, with at least 30 days of abstinence required. Current HTP/EC use is the union of the EC item and the HTP item. The HTP/EC group comprises former smokers currently using either product; the no-product group comprises former smokers using neither. Short-term and long-term strata are defined at 5 years of cessation (short-term less than 5 years) to mirror the two-screening classifier described by Kim et al.

### Product classification.

The NHIS e-cigarette item is reported by Kim et al. only in English translation, so the ambiguity in their instrument cannot be read directly. It can be read indirectly: the authors themselves note that other publications describe the same questionnaire-derived exposure by a broader umbrella term, citing Choi et al., whose account of the identical item states that it does not differentiate heated tobacco products from nicotine vapor products. An independent KNHANES analysis agrees: Lee et al.^9^ caution that some 2018 cigarette-and-HTP dual users may have been misclassified as cigarette-and-EC dual users. In the raw 2018 file, 205 respondents reported current HTP use on the explicit HTP checklist, but only 171 reported current use on the umbrella e-cigarette item; asking about HTP directly therefore identifies 34 more users than the umbrella question captures, indicating that an umbrella item of the kind the NHIS used undercounts HTP rather than subsuming it. Because the NHIS item’s wording and responses are unpublished, the exposed group’s product mix can be bounded but not measured, and the two natural readings of a single e-cigarette question bound it narrowly. If the item captured only respondents who identified with the narrow e-cigarette term, as the separate 2018 KNHANES e-cigarette item did, the exposed group would comprise only those responders: of the 43 KNHANES switchers, 18, of whom 9 (50%) also used HTP, the 25 HTP-only switchers being classified into the comparator. If instead the item did not differentiate HTP from vapor, as the authors’ own cited source describes, it would capture all product users and the composition would match the direct KNHANES measurement, 79% HTP. HTP use in the exposed group therefore falls between 50% and 79% under these two readings. An HTP frequency item exists only from 2019, so a daily-use restriction is not definable for HTP/EC in 2018.

### Cumulative exposure.

Pack-years were derived for the screening-eligible restriction as the average number of cigarettes per day among former smokers (BS6_3, divided by 20) multiplied by total smoking duration in years (BS6_2, recorded in months and verified to equal 12 × BS6_2_1 + BS6_2_2 in all 1,352 records with valid components). Codes of 88 and above were treated as missing; pack-years were derivable for 1,345 of 1,348 respondents. The screening-eligible subgroup is defined as in Kim et al., aged 50 to 80 years with at least 20 pack-years.

### Sample size and exclusions.

Exact numbers are reported at each step. For the 2018 baseline the flow is 7,992 total records, then 6,489 adults aged 19 and older, then 1,356 former smokers (BS3_1 = 3), then 1,355 with a valid cessation duration, then 1,348 with at least 30 days of abstinence, of whom 43 were current HTP/EC users and 1,305 used neither. Two exclusions apply: one former smoker with a refusal or missing cessation code (BS6_4 at least 9000) and seven reporting fewer than 30 days of abstinence, none of whom were HTP/EC users. In each wave examined the 30-day rule excludes fewer than ten respondents (seven in 2018 and five in 2019) and leaves every reported median unchanged. Per-wave and per-group exact n values are listed in Supplementary Tables S1 and S3 and in Figures 1 and S3.

### Weighting and external benchmark.

The comparisons reported below are internal relative comparisons (product composition, cessation-duration contrasts and age-matched residuals) that do not depend on population projection, and are therefore computed unweighted on the analytic sample. Where an absolute national prevalence is at issue the survey design must be applied, and we benchmark against a peer-reviewed KNHANES analysis. Applying the KNHANES sampling weights to the 2019 file, current HTP use under the standard definition (daily or occasional) is 8.6% among adult men, matching the 8.8% reported by Lee et al.^9^ for the same wave using the same definition; female HTP use is 1.5% in both. The exposure rule used by Kim et al., which counts only daily use, yields 5.4% among men against the same weights, undercounting the published national estimate by approximately one third. Weighted male liquid-EC use in 2019 was 4.2%, less than male HTP use, consistent with HTP being the predominant novel product in the exposed group. These weighted figures are reported only to establish that the corrected HTP definition reproduces the national benchmark and that the daily-use restriction departs from it; the within-sample contrasts elsewhere are unweighted, as noted, and do not depend on the benchmark.

### Statistical comparisons.

Cessation duration is strongly right skewed and bounded at zero, so we did not assume normality: the median is used as the measure of center and the interquartile range (IQR) as the measure of spread, and all comparisons use rank-based or robust methods that make no normality or equal-variance assumption. Means are shown alongside medians in Supplementary Table S3 only for comparability with Kim et al.; given the skew they are not the primary summary. Cessation-duration distributions (Figure 1A and 1B) are compared with the two-sample Kolmogorov–Smirnov test, the two-sided Mann–Whitney rank-sum test, and the probability of superiority, the proportion of cross-group pairs in which the HTP/EC switcher had quit more recently (ties counted as one half). The age-matched comparison restricts the no-product group to those former smokers for whom at least one HTP/EC switcher falls within 2 years of age (n = 941 of 1,305 in 2018) and compares that matched pool with all HTP/EC switchers using the same measures; the residual gap is the median cessation of the matched pool minus the median among HTP/EC switchers. In Figure 1C the age–duration relationship is summarized per group by Theil–Sen robust regression, and the difference in slopes is tested with an age-by-group interaction term. The dashed reference line in that panel indicates a cessation duration of 10 years, the interval between the 2008 introduction of e-cigarettes in Korea and the 2018 baseline. All tests are two-sided and unweighted, consistent with the internal-comparison framing above. Exact P values are reported where estimable and as P < 0.001 otherwise.

### Statistical reporting.

Tests used are the two-sample Kolmogorov–Smirnov test, the Mann–Whitney rank-sum test, Theil–Sen robust regression, and an ordinary-least-squares age-by-group interaction; all are two-sided. Our models contain age, group and their interaction and no other covariates. No covariate screening or selection was performed and no covariate was tested and omitted. Normality is not assumed anywhere; the rank tests assume only independent observations, and the interaction is an ordinary-least-squares contrast of slopes under pooled variance, for which we report the coefficient, standard error, degrees of freedom and confidence interval. No multiplicity adjustment is applied: the comparisons are descriptive contrasts of a single quantity, cessation duration, reported in full rather than selected on significance, and the argument rests on the size of the separation rather than on any individual P value. Applying a Bonferroni correction across the tests reported in Supplementary Tables S3 and S5 would leave every conclusion unchanged except the age-by-group interaction, on which we do not rely. KNHANES is a stratified multistage cluster sample, with design variables kstrata, psu and wt_itvex; the internal contrasts are computed unweighted and without a clustering adjustment, so the P values reported here do not account for design effects and should be read as descriptive. The weighted prevalence benchmark above is the only quantity for which the design is applied. All observations are distinct respondents measured once.

### Software and reproducibility.

Analyses were initially performed in Python with statsmodels (interaction model) and then manually reverified in Excel. EC and HTP definitions and derivations are shown in Supplementary Figures S1 and S2.

## Results

### Analytic sample

The KNHANES 2018 analytic sample comprised 1,348 former smokers aged 19 years and older with a valid cessation duration of at least 30 days. Of these, 43 currently used a heated tobacco product or an e-cigarette (the HTP/EC group) and 1,305 used neither (the no-product group). Median age was 39 years in the HTP/EC group.

### Exposure composition: non-combusted product mix

Among HTP/EC-using former smokers in 2018, 58% reported HTP use without e-cigarette use, 21% reported both HTP and e-cigarette use, and 21% reported e-cigarette use without HTP use (Table 1). Overall, 79% reported any HTP use. Product-using former smokers were a minority of product users overall: 222 current smokers also reported HTP or EC use, giving a dual-user to complete-switcher ratio of 5.2 to 1.

### Time since cessation

Former smokers using HTP or EC had quit smoking far more recently than former smokers using no product. Median cessation duration was 0.7 years (approximately eight months; IQR 0.25 to 1.5) in the HTP/EC group, against 12.0 years (IQR 5.0 to 20.0) in the no-product group. The two distributions barely overlapped (Figure 1A; Kolmogorov–Smirnov D = 0.76, P < 0.001), and in 92% of cross-group pairwise comparisons the HTP/EC switcher had quit more recently. For the 2018 overall contrast, Mann–Whitney U = 4,391 (n_1_ = 43, n_2_ = 1,305; P = 4.2 × 10^−21^) and Kolmogorov–Smirnov D = 0.758 (P = 3.3 × 10^−25^).

### The separation persists within cessation strata

Stratifying at five years of cessation, as the two-screening classifier described by Kim et al. does, narrowed but did not remove the imbalance. Within the short-term (<5 year) stratum the cessation-duration distributions remained separated (Figure 1B; D = 0.34, P < 0.001), and the HTP/EC switcher had quit more recently in 73% of cross-group pairs (U = 3,480; n_1_ = 41, n_2_ = 311; P = 2.1 × 10^−6^; D = 0.341, P = 0.0003). Within-stratum medians were 0.6 years (n = 41) for HTP/EC against 2.0 years (n = 311) for no-product in the short-term stratum. The long-term HTP/EC cell in 2018 contains only two respondents and is reported for completeness only; the long-term contrast is more securely read from 2019, where the cell contains nine respondents and the same ordering holds (6.0 against 16.0 years).

### Age adjustment does not stand in for cessation duration

Among former smokers using no product, cessation duration rose with age (Theil–Sen slope +0.30 years per year of age); among HTP/EC users it was flat across the age range (−0.01) (Figure 1C). The age-by-group interaction coefficient was -0.359 (SE 0.166; t = −2.16; df = 1,344; 95% CI −0.686 to −0.033; P = 0.031). Restricting the no-product group to respondents age-matched to within two years of an HTP/EC switcher (n = 941 of 1,305) left a residual gap of 9.3 years between the group medians. In 2018 the longest-abstinent HTP/EC switcher had quit 10.0 years earlier, and 53.3% of no-product quitters, and 70.0% of those in the long-term stratum, had quit longer ago than any switcher in the sample.

### The age 50 and older subgroup

Kim et al. report a sensitivity analysis restricted to participants aged 50 years and older, the stratum in which most lung cancer arises and which corresponds to screening-eligible age. Restricting KNHANES 2018 to respondents aged 50 to 80 does not attenuate the cessation-duration imbalance; it widens it. Among no-product quitters in that age band (n = 858), median cessation duration was 15.5 years (IQR 7.0 to 25.0; mean 17.6), against 12.0 years in the unrestricted sample. Only four HTP/EC switchers were aged 50 or older, with cessation durations of 0.25, 0.25, 1.0 and 2.0 years; none had quit more than two years before the survey.

The four exposed observations are far too few to estimate a median with any precision, and we do not treat them as one. They are, however, sufficient to show that the exposed cell does not migrate up the cessation-duration axis with age, which is what the Theil–Sen fits in Figure 1C describe for the sample as a whole. Taking the exposed value in this age band to be approximately one year, which is consistent both with those four observations and with the flat age–duration relationship among product users, the residual gap against age-restricted no-product quitters is approximately 14.5 years, compared with 11.3 years in the unrestricted sample. In this age band 93.0% of no-product quitters had quit more than one year before the survey, 63.4% more than ten years, and 31.7% more than twenty years; the switcher had quit more recently in 95.2% of cross-group pairs, against 92% unrestricted.

Kim et al. define their high-risk subgroup by screening eligibility, aged 50 to 80 years with at least 20 pack-years, and report a larger estimate there than in the cohort overall. KNHANES permits that definition to be reconstructed: smoking duration is recorded in months (BS6_2) and average cigarettes per day among former smokers in BS6_3, giving pack-years as the product of packs per day and years smoked. Applying both criteria leaves 421 no-product quitters and 3 HTP/EC switchers. Cumulative exposure is well balanced between them (mean 37.6 and 37.7 pack-years; median age 67 and 66), which is what the restriction is designed to achieve. Time since cessation is not: median cessation was 11.0 years (IQR 5.0 to 20.0) among no-product quitters, against 0.25, 1.0 and 2.0 years for the three switchers. Ninety percent of the no-product group had quit more than a year before the survey, 51.5% more than ten years, and 86.0% had quit longer ago than the most abstinent switcher in the cell.

Restricting to older participants therefore selects, on the comparison side, for progressively more remote cessation while leaving the exposed side unchanged. Under the age criterion alone the residual gap widens from 11.3 to approximately 14.5 years; adding the pack-year criterion, which removes the lightest and shortest-duration smokers from the comparison group, returns it to approximately 10 years. Neither restriction brings the two groups closer together on time since quitting than the unrestricted comparison. A restriction of this kind concentrates outcome events and equalizes cumulative exposure, but does not act as a proxy adjustment for cessation recency.

### Reproducibility across waves

The overall contrast and the long-term stratum contrast reproduce in every KNHANES wave from 2018 to 2023, and the age-matched residual ranges from 9 to 11 years across those waves (Supplementary Table S3). In 2019, the wave in which the product items are explicit, median cessation was 2.0 years in the HTP/EC group (n = 101) against 12.0 years in the no-product group (n = 1,326), with an age-matched residual of 9.0 years.

## Discussion

In a nationally representative survey of the same population and the same calendar year as the NHIS screening cohort analyzed by Kim et al., Korean former smokers using non-combusted nicotine products differed from other former smokers in two respects that bear directly on how such comparisons should be read. First, they were predominantly HTP users: 79% reported any HTP use, and only 21% reported e-cigarette use without HTP use. Second, they had stopped smoking approximately a decade more recently, a difference that survived stratification at five years and exact age matching. Neither quantity is recorded in the NHIS screening instrument.

### Product composition

Heated tobacco products entered the Korean market shortly before the 2018 baseline wave and were widely used by 2018.^6–9^ The authors of the cohort study acknowledge that the 2018 NHIS instrument had no separate HTP item, creating potential misclassification between liquid e-cigarettes and HTPs.^3^ KNHANES, fielded the same year, did distinguish HTP use from the e-cigarette item, and shows that the great majority of product-using former smokers were using HTPs. Depending on how respondents interpreted the NHIS e-cigarette item, HTP users may have comprised between half and four fifths of the exposed group. An association estimated from such a group should not be read as product-specific evidence about liquid e-cigarettes. This bounding also indicates the direction of a design improvement: from 2019 the KNHANES instrument separates the two product classes explicitly, and cohort analyses using later waves can define exposure accordingly.

### Time since cessation

Lung cancer risk remains strongly influenced by recency of cigarette smoking and declines only gradually after cessation.^1,2^ An analysis that combines recent switchers with long-term former smokers may therefore attribute residual risk from recent cigarette smoking to post-cessation product use. Kim et al. could not adjust directly for this variable because the NHIS did not record a quit date.^3^ KNHANES measures time from the last cigarette to the month, and shows that this unmeasured variable differed substantially between the groups being compared.

Stratified and age-adjusted analyses do not by themselves resolve this. The short-term and long-term categories available in the cohort were derived from smoking status at two screening waves rather than from exact quit dates, and the long-term category has no lower bound on cessation duration. In KNHANES, stratification at five years narrowed the imbalance but left the distributions separated. Age adjustment is likewise an incomplete proxy: cessation duration rises with age among quitters using no product but is flat among product users, so matching on age does not equalize time since quitting, leaving a residual of about nine years. This has a direct bearing on the screening-eligible subgroup, in which Kim et al. report their largest estimate. Restricting KNHANES to ages 50 to 80 raises median cessation among no-product quitters from 12.0 to 15.5 years, while the few product users in that band had all quit within two years; adding the 20 pack-year criterion returns the gap to about ten years. Cumulative exposure is closely matched under the full restriction, as intended, but time since quitting is not, and 86% of the comparison group had quit longer ago than the most abstinent product user in that cell. The subgroup contributing the most outcome information is therefore not one in which the cessation-duration imbalance has been resolved. Eligibility criteria of this kind select on cumulative exposure and age; they do not substitute for adjustment on cessation recency.

### Interaction with sparse outcome cells

Measurement imprecision of this kind matters more when the estimate it feeds is thinly supported. In the cohort, the long-term quitter comparison represents approximately two thirds of quitter person-time but only 20% of switcher person-time, and rests on 16 lung cancer events among switchers against approximately 11,420 in the reference group (Supplementary §5). Sixteen events adjusted for ten covariates is approximately 1.6 events per covariate, below the ten events per covariate conventionally treated as a minimum for stable Cox estimation.^5^ Where the exposed and reference groups also differ substantially in cessation duration and product-use history, as the present analysis indicates they do, estimates of this structure may be sensitive to model specification and to residual confounding.

A related question of emphasis concerns dual users. In the authors’ 2024 conference abstract, current smokers with and without e-cigarette use were separate exposure groups; in the published paper they are combined into a single current-smoker reference, and the dual-use comparison appears in the supplement.^13^ There, compared with exclusive cigarette smokers, dual users had lower lung cancer incidence (aHR 0.78, 95% CI 0.72 to 0.85), lower lung cancer death (0.66, 0.55 to 0.79) and lower all-cause mortality (0.74, 0.71 to 0.78).^3^ That contrast rests on 578 exposed events against 71 for complete switchers and has the narrowest confidence interval of any product comparison in the study. We draw no causal inference from it, since it is open to the same confounding described above; the observation is that the best-powered product comparison in the study is reported in the supplement while a 16-event contrast is emphasized in the abstract.

### Relation to previously raised concerns

The Shields commentary raised three concerns about this literature: short follow-up relative to reverse causation, poorly characterized exposure with unverified HTP use, and small exposed cells relative to the effect sizes claimed.^10^ Reverse causation cannot be quantified from the published data. The present analysis addresses the second concern by measuring the product mix directly rather than inferring it, and bears on the third by showing that the groups whose comparison generates the sparse estimate are separated on an unmeasured variable strongly related to the outcome.

### Limitations

KNHANES is a cross-sectional survey and contains no lung cancer outcomes; it cannot reproduce, correct or refute any hazard ratio, and no estimate reported here is a re-estimate of any published association. The KNHANES and NHIS samples are distinct, and although both are drawn from the same national adult population in the same years, we cannot demonstrate that the exposure composition observed in one holds exactly in the other; the NHIS item wording and response distribution are unpublished, which is why product composition is bounded rather than measured. The HTP/EC cell is small in 2018 (n = 43), and smaller still within the long-term stratum (n = 2) the age 50 and older band (n = 4) and the screening-eligible subgroup (n = 3); no median from those cells should be read as a stable estimate, and the age-restricted comparison is reported as a bound on where the exposed group sits rather than as an estimate of its center. We therefore report the 2019 wave, with n = 101 and nine long-term respondents, alongside it, and the contrast reproduces through 2023. Product use is self-reported and measured at a single time point, so continuity of use cannot be assessed. Internal contrasts are unweighted and unadjusted for the survey design, and the P values should be read as descriptive; the weighted benchmark is reported separately and reproduces a published national estimate. Finally, cessation duration is self-reported and subject to recall error, though such error would have to be both large and strongly differential to account for a separation of the magnitude observed.

### Conclusions

Nothing in this analysis shows that switching to e-cigarettes or heated tobacco products is safe, and we do not claim that it does. What it shows is that in the Korean population at the relevant time, the group that a single undifferentiated e-cigarette question would classify as post-cessation product users was composed largely of HTP users and consisted disproportionately of very recent quitters, while the comparison group consisted largely of long-term former smokers. Cohort estimates built on that contrast should be interpreted with explicit attention to both quantities.

Better instruments and designs exist, including continuous cessation duration in later NHIS waves, product-specific items from 2019 onward, repeated assessment of switching and relapse, and designs that match on time since quitting rather than dichotomizing it. Given the long latency of lung cancer and the rapid evolution of product use patterns, resolving whether post-cessation use of a non-combusted product independently affects lung cancer risk relative to quitting outright will require precise longitudinal exposure and cessation-history measurement. Until such studies are available, the reported association is best treated as a signal requiring replication with more precise exposure and cessation-history measurement rather than as definitive evidence.

## Supplementary Material

Supplement 1

## Figures and Tables

**Figure 1.**
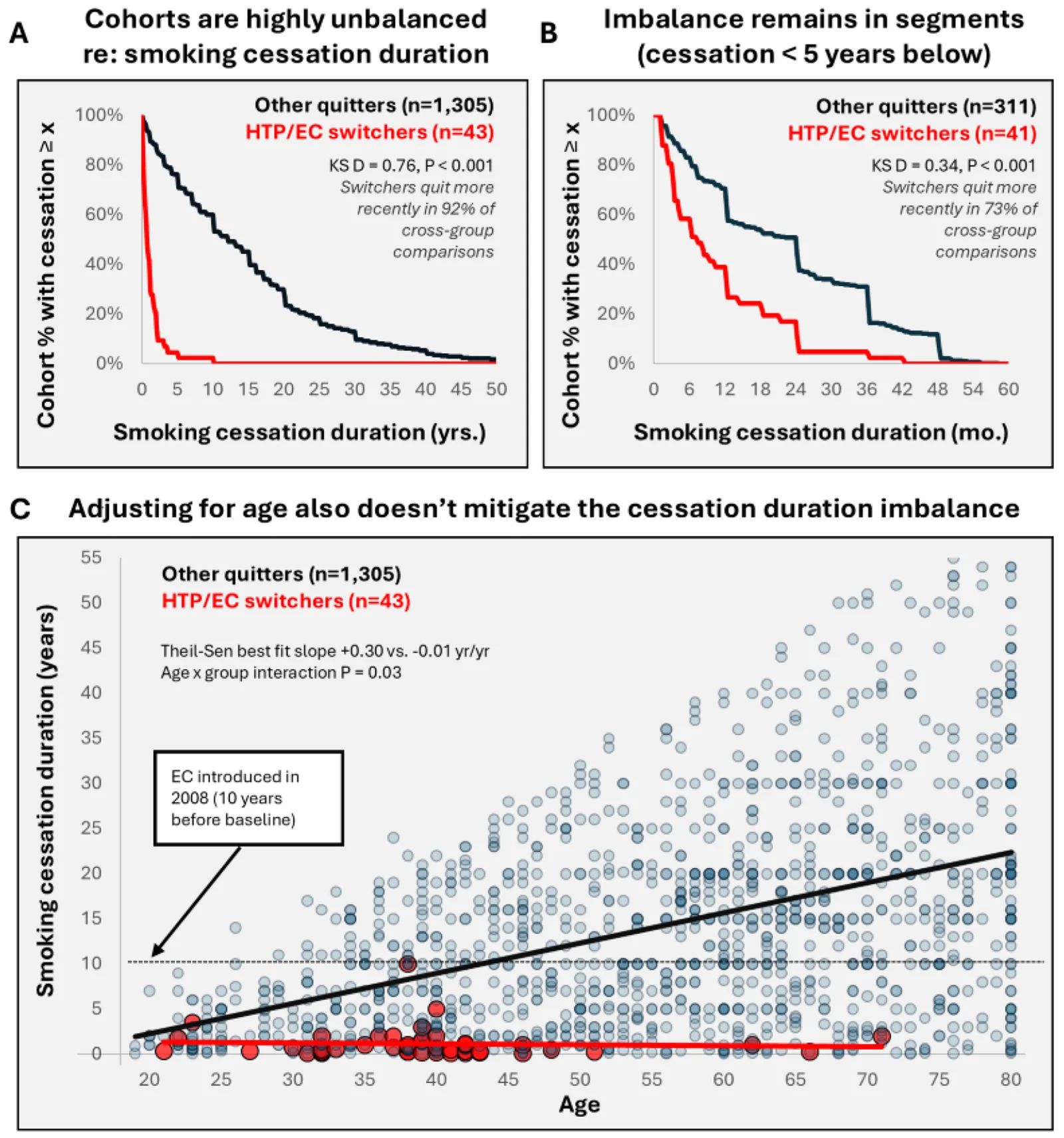
Smoking cessation duration among former smokers, KNHANES 2018. HTP/EC switchers (red; n = 43) vs. quitters by other means (blue; n = 1,305). (A) Complementary cumulative distribution across years for all former smokers (Kolmogorov–Smirnov D = 0.76; switchers quit more recently in 92% of cross-group comparisons; P < 0.001). (B) The same in months within the short-term (<5-year) stratum (D = 0.34, 73%, P < 0.001). (C) Age vs. cessation duration with per-group Theil–Sen fits; the dashed line marks cessation beginning in 2008, when e-cigarettes first went on sale in Korea; HTPs reached the market in June 2017.

**Table 1.** Exposure composition among HTP/EC users, KNHANES 2018

Metric	KNHANES 2018
**A. Combustible-cigarette status among ever-smokers currently using HTP or EC**	
Dual users (current combustible + HTP/EC), n	222
Complete switchers (former combustible + HTP/EC), n	43
Total, n	265
Complete switchers, %	16.2%
Dual-user : switcher ratio	5.2 : 1
**B. Product type among HTP/EC-using former smokers, n (%)**	
HTP only	25 (58%)
HTP, liquid-EC status unconfirmed	9 (21%)
Liquid EC only	9 (21%)
Total HTP/EC former smokers, n	43

Panel A: switchers are the analytic sample (former smokers with a valid cessation duration of at least 30 days); dual users are current smokers reporting HTP or EC use, for whom no cessation filter applies. Panel B denominator: HTP/EC-using former smokers in that analytic sample. In 2018 the e-cigarette item was an undifferentiated umbrella, so a positive response alongside HTP confirms HTP use but not liquid-EC use; that cell is therefore reported as HTP with unconfirmed liquid-EC status, and confirmed dual use is not definable for 2018.

## Data Availability

The KNHANES public-release microdata (2018 to 2023) are freely available from the Korea Disease Control and Prevention Agency at https://knhanes.kdca.go.kr.
