# Supplementary material for: Product mix and time since cessation among Korean former smokers using non-combusted nicotine products: a KNHANES analysis with implications for lung cancer risk comparisons
Source: medRxiv. 2026 Aug 13:2026.08.11.26360188. Preprint. [Version 1] doi: 10.64898/2026.08.11.26360188 (PMC13640878; doi:10.64898/2026.08.11.26360188)
Supplement: Supplementary file 1 [file media-1.pdf]

Supplementary Appendix

Product mix and time since cessation among Korean former smokers using non-combusted nicotine products: a KNHANES analysis with implications for lung cancer risk comparisons

Preface

This appendix accompanies the main paper and reports in full the derivations, per-wave tables and sensitivity analyses summarized there. Sections 1 to 3 document the KNHANES instrument, the product definitions and the exposure composition; sections 4 to 7 report the cessation-duration contrasts, the age-matched residuals, and the sensitivity analysis restricting the exposed group to respondents affirming the umbrella e-cigarette item. Some material is repeated from the main text so that this appendix can be read on its own. Throughout, the study of Kim et al. is referred to as the motivating cohort; KNHANES contains no lung cancer outcomes and no analysis here re-estimates any published association.

1. Methods

Data source. All analyses in this appendix use the public-release microdata of the Korea National Health and Nutrition Examination Survey (KNHANES) from 2018 to 2023 [5]. The KNHANES is a nationally representative, annually fielded cross-sectional survey conducted by the Korea Disease Control and Prevention Agency [4], drawing on the same adult population and the same calendar years as the National Health Insurance Service (NHIS) screening cohort analyzed by Kim et al. [1]. Kim et al. neither analyze nor cite KNHANES; we use it because it is the national reference source for tobacco use surveillance in Korea. In Kim's 2018 baseline year, it records the two quantities for which the NHIS screening item does not (time quit smoking and product used). The NHIS asked a single question about "e-cigarette" use in the previous month, with no way to separate the products or to date the smoking quit point.

The KNHANES 2018 questionnaire included a first question asking about current e-cigarette use (questionnaire item Q11-1; variable BS12_2). Here, the term e-cigarette was generic and not specified to be "liquid nicotine-containing". It was then followed by a checklist (questionnaire item Q12-1; heated tobacco option, variable BS12_47) asking whether certain products were currently used, excluding e-cigarettes, and one of the choices was heated tobacco products, defined with specific examples (IQOS, glo). Therefore, we can deduce that someone who responded affirmatively to question 12 but negatively to question 11 was exclusively using HTP. Likewise, yes to 11 and no to 12 would correspond to exclusive liquid EC use. Yes to both is ambiguous and could mean either the dual use of both or the exclusive use of HTP. See Figure S1 for the questions asked in Korean.

From KNHANES 2019 onward, the questions are asked more specifically. Question 4-1 asks about current HTP use and provides specific examples. This is followed by question 5-1, which asks about liquid nicotine EC use (see Figure S2).

Cessation duration was recorded in months (BS6_4 in 2018), whereas the NHIS captured only smoking status at two screening dates five years apart. From 2019, the KNHANES standardized the HTP question into daily, occasional, and former categories with frequency of use also asked, refining the product distinction it already drew in 2018 rather than introducing one.

Population and definitions. Analysis is restricted to adults aged 19 years and older. The smoking-status item is administered only to respondents who have smoked, so BS3_1 = 3 identifies former smokers without a further lifetime-use filter. Current smokers are BS3_1 in{1 (daily), 2 (occasional)}; former smokers are BS3_1 = 3. Cessation duration is BS6_4, recorded in months and divided by 12, excluding codes of 8888 and above, with at least 30 days of abstinence required. Current HTP/EC use is the union of the EC item and the HTP item. The HTP/EC group comprises former smokers currently using either product; the no-product group comprises former smokers using neither. Short-term and long-term strata are defined at 5 years of cessation (short-term less than 5 years) to mirror the two-screening classifier described by Kim et al.

Product classification. The NHIS e-cigarette item is reported by Kim et al. only in English translation, so the ambiguity in their instrument cannot be read directly. It can be read indirectly: the authors themselves note that other publications describe the same questionnaire-derived exposure by a broader umbrella term, citing (their reference 47) Choi et al., whose account of the identical item states that it does not differentiate heated tobacco products from nicotine vapor products. An independent KNHANES analysis agrees: Lee et al. [9] caution that some 2018 cigarette-and-HTP dual users may have been misclassified as cigarette-and-EC dual users. In the raw 2018 file, 205 respondents reported current HTP use on the explicit HTP checklist, but only 171 reported current use on the umbrella "e-cigarette" item; thus, asking about HTP directly identifies 34 more users than the umbrella question captures, an umbrella item of the kind the NHIS used undercounts HTP rather than subsuming it. Because the NHIS item's wording and responses are unpublished, the exposed group's product mix can be bounded but not measured, and the two natural readings of a single e-cigarette question bound it narrowly. If the item captured only respondents who identified with the narrow e-cigarette term, as KNHANES's separate 2018 e-cigarette item did, the exposed group would comprise only those responders: of the 43 KNHANES switchers, 18, of whom 9 (50%) also used HTP, the 25 HTP-only switchers being misclassified into the comparator. If instead the item did not differentiate HTP from vapor, as the authors' own cited source (Choi et al.) describes, it would capture all product users, and the composition would match the direct KNHANES measurement, 79% HTP (Table S1, Panel B). The HTP use in the exposed group therefore falls between 50% and 79% under these two readings. An HTP frequency item exists only from 2019; thus, a daily use restriction is not definable for HTP/EC in 2018.

Sample size and exclusions. Exact numbers are reported at each step; for the 2018 baseline, the flow is 7,992 total records, then 6,489 adults aged 19 and older, then 1,356 former smokers (BS3_1 = 3), then 1,355 with a valid cessation duration, and then 1,348 with at least 30 days of abstinence, of whom 43 were current HTP/EC users and 1,305 used neither. Two exclusions apply: one former smoker with a refusal or missing cessation code (BS6_4 at least 9000) and seven reporting fewer than 30 days of abstinence (none of whom were HTP/EC users). In each wave examined, the 30-day rule excludes fewer than ten respondents (seven in 2018 and five in 2019) and leaves every reported median unchanged. The per-wave and per-group exact n values are listed in Tables S1 and S3 and in Figures 1 and S3.

Weighting and external benchmark. The comparisons reported below are internal relative comparisons (product composition, cessation-duration contrasts, and age-matched residuals) that do not depend on population projection and are therefore computed unweighted on the analytic sample. Where an absolute national prevalence is at issue, the survey design must be applied, and we benchmark against a peer-reviewed KNHANES analysis. Applying the KNHANES sampling weights to the 2019 file, the current HTP use under the standard definition (daily or occasional) is 8.6% among adult men, matching the 8.8%

reported by Lee et al. [9] for the same wave using the same definition; female HTP use is 1.5% in both. The exposure rule used by Kim et al., which counts only daily use, yields 5.4% among men against the same weights, undercounting the published national estimate by approximately one-third. Weighted male liquid-EC use in 2019 was 4.2%, which is less than male HTP use, which is consistent with HTP being the predominant novel product in the exposed group. These weighted figures are reported only to establish that the corrected HTP definition reproduces the national benchmark and that the daily use restriction departs from it; the within-sample contrasts elsewhere in this appendix are unweighted, as noted, and do not depend on the benchmark.

Statistical comparisons. Cessation duration is strongly right skewed and bounded at zero; thus, we did not assume normality: the median is used as the measure of center, and the interquartile range (IQR) is used as the measure of spread, and all comparisons use rank-based or robust methods that make no normality or equal-variance assumption. In 2018, the median [IQR] cessation duration was 0.7 (0.25–1.5) years among HTP/EC switchers versus 12.0 (5.0–20.0) years among no-product quitters. Means are shown alongside medians in Table S3 only for comparability with Kim et al.; given the skew, they are not the primary summary. Cessation-duration distributions (Figure 1A and 1B) are compared with the two-sample Kolmogorov–Smirnov test, the two-sided Mann–Whitney rank-sum test, the probability of superiority, the proportion of cross-group pairs in which the HTP/EC switcher had quit more recently (ties counted as one half). The age-matched comparison restricts the no-product group to those former smokers for whom at least one HTP/EC switcher falls within 2 years of age (n = 941 of 1,305 in 2018), and compares that matched pool with all HTP/EC switchers using the same measures; the residual gap is the median cessation of the matched pool minus the median among HTP/EC switchers. In Figure 1C, the age–duration relationship is summarized per group by Theil–Sen robust regression, and the difference in slopes is tested with an age–by–group interaction term. The dashed reference line in that panel indicates a cessation duration of 10 years, the interval between the 2008 introduction of e-cigarettes in Korea and the 2018 baseline. All tests are two-sided and unweighted, which is consistent with the internal-comparison framing above. Exact P values are reported where estimable and as P values less than 0.001 otherwise.

Statistical reporting. Tests used are the two-sample Kolmogorov-Smirnov test, the Mann-Whitney rank-sum test, Theil-Sen robust regression, and an ordinary-least-squares age-by-group interaction; all are two-sided. Covariates: our own models contain age, group and their interaction and no others. No covariate screening or selection was performed and no covariate was tested and omitted. Assumptions: normality is not assumed anywhere, as set out above; the rank tests assume only independent observations, and the interaction is an ordinary-least-squares contrast of slopes under pooled variance, for which we report the coefficient, standard error, degrees of freedom and confidence interval. Multiple comparisons: no multiplicity adjustment is applied. The comparisons are descriptive contrasts of a single quantity, cessation duration, reported in full rather than selected on significance, and the argument rests on the size of the separation rather than on any individual P value. Applying a Bonferroni correction across the tests reported in Tables S3 and S5 would leave every conclusion unchanged except the age-by-group interaction, on which we do not rely (§7). Survey design: KNHANES is a stratified multistage cluster sample, with design variables kstrata, psu and wt_itvex. The internal contrasts are computed unweighted and without a clustering adjustment, so the P values reported here do not account for design effects and should be read as descriptive; the weighted prevalence benchmark above is the only quantity for which the design is applied. All observations are distinct respondents measured once; no respondent is measured repeatedly.

Test statistics and effect sizes. For the 2018 overall contrast, Mann-Whitney U = 4,391 (n1 = 43, n2 = 1,305; P = 4.2 x 10-21) and Kolmogorov-Smirnov D = 0.758 (P = 3.3 x 10-25). Within the short-term stratum, U = 3,480 (n1 = 41, n2 = 311; P = 2.1 x 10-6) and D = 0.341 (P = 0.0003). The age-by-group interaction coefficient is -0.359 (SE 0.166; t = -2.16; df = 1,344; 95% CI -0.686 to -0.033; P = 0.031). Effect sizes are the Kolmogorov-Smirnov D, the probability of superiority computed as U divided by the product of the two group sizes with ties counted as one half, the Theil-Sen slopes, and the age-matched residual gap in years. No Bayesian analysis was performed.

Software and reproducibility. Analyses were initially performed in Python and statsmodels (interaction model) and then manually reverified in Excel. EC and HTP definitions and derivations are shown in Figures S1 and S2.

Data and code availability. The KNHANES public-release microdata (2018 to 2023) are freely available from the Korea Disease Control and Prevention Agency at https://knhanes.kdca.go.kr.

Disclosure of computational tooling. Preliminary analysis of the KNHANES public-release files was performed with the assistance of Claude (Anthropic). The authors generated the figures and verified the data and statistical tests using Excel.

2. Definitions of EC and HTP

In 2018, the KNHANES asked about current EC use, without specifying that it pertained to liquid nicotine, followed by a specific HTP term. In 2019, the KNHANES asked about current HTP use, using a specific HTP term, followed by current EC use, using a specific term (liquid nicotine).

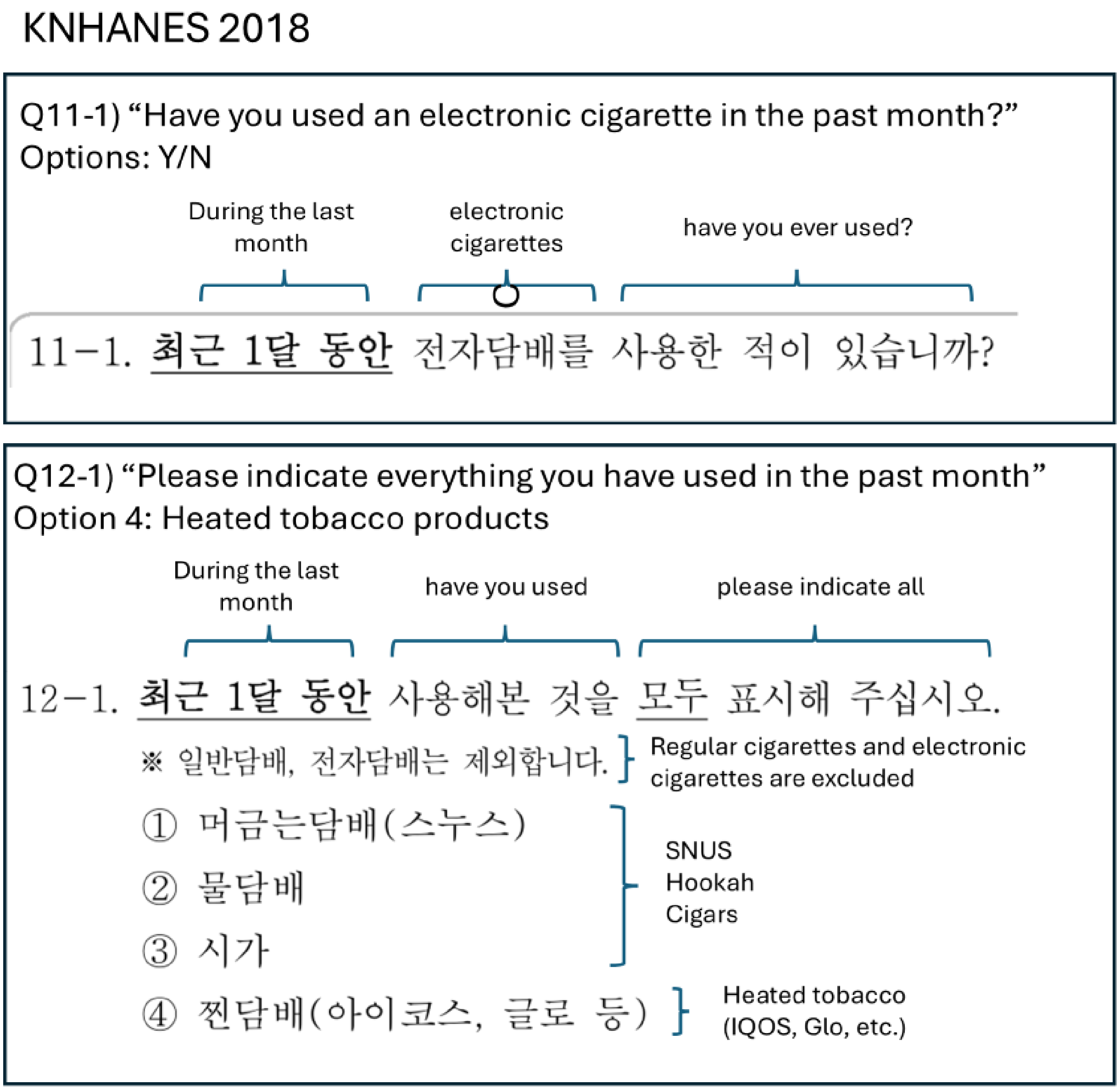

Figure S1. E-cigarette and HTP current use questions, KNHANES 2018. *In 2018, participants were first asked about e-cigarette use, followed by a question on heated tobacco products. The e-cigarette term did not specify liquid nicotine, while the HTP included specific brands as examples (IQOS, Glo).*

KNHANES 2019 onward

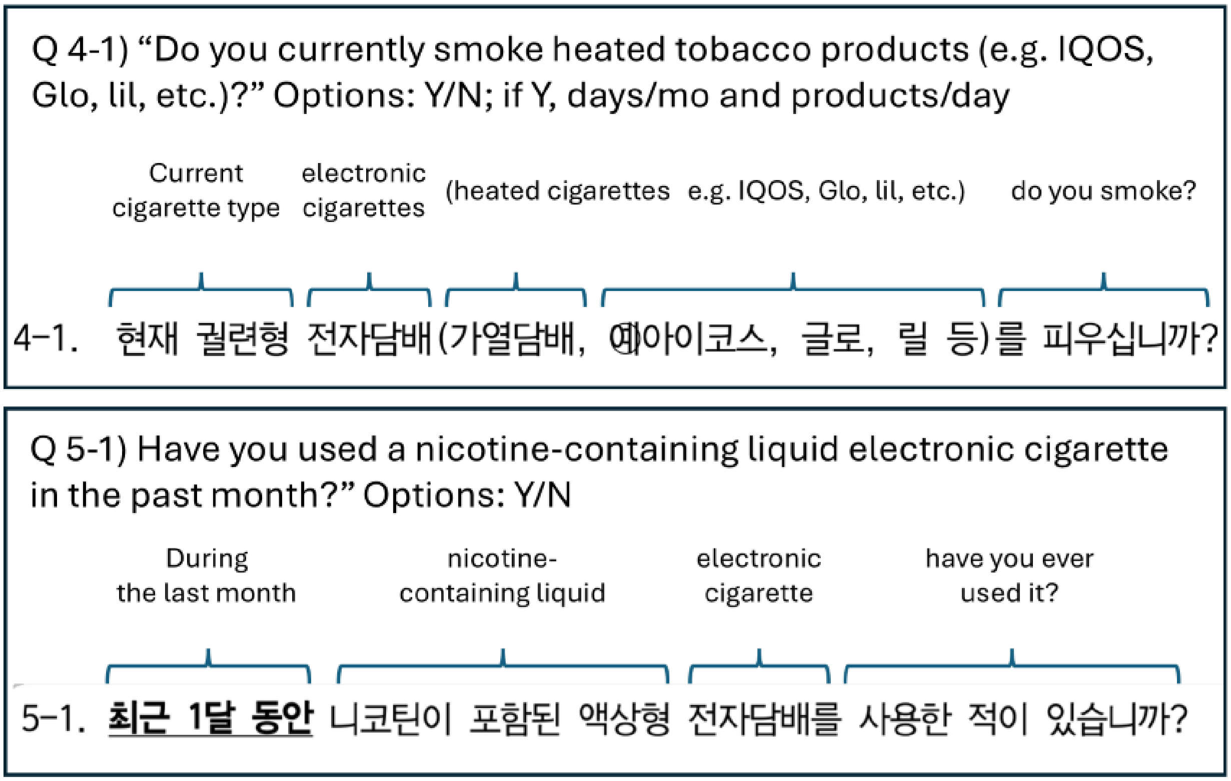

Figure S2. E-cigarette and HTP current use questions, KNHANES 2019 onward. *In 2019, participants were first asked about HTP use, followed by a question on nicotine-containing liquid e-cigarettes. The e-cigarette term specified nicotine-containing liquid EC, and the HTP included specific brands as examples (IQOS, Glo, lil).*

3. Exposure composition

Table S1. Exposure composition among HTP/EC users, KNHANES 2018 and 2019

Metric	KNHANES 2018	KNHANES 2019
A. Combustible-cigarette status among ever-smokers currently using HTP or EC		
**Dual users (current combustible + HTP/EC), n**	**222**	**214**
**Complete switchers (former combustible + HTP/EC), n**	**43**	**101**
Total, n	265	315
Complete switchers, %	16.2%	32.1%
**Dual-user : switcher ratio**	**5.2 : 1**	**2.1 : 1**
B. Product type among HTP/EC-using former smokers, n (%)		
**HTP only**	**25 (58%)**	**62 (61%)**
**HTP, liquid-EC status unconfirmed**	**9 (21%)**	**N/A**
**HTP and liquid EC (confirmed dual)**	**N/A**	**14 (14%)**
**Liquid EC only**	**9 (21%)**	**25 (25%)**
Total HTP/EC former smokers, n	43	101
C. Use frequency among current HTP users		
Former-smoker HTP users: daily, n (%)	not measured	67 (88%)
Former-smoker HTP users: occasional, n (%)	not measured	9 (12%)

Panel A: switchers are the analytic sample (former smokers with a valid cessation duration of at least 30 days); dual users are current smokers reporting HTP or EC use for whom no cessation filter applies. Panel B denominator: HTP/EC-using former smokers in that analytic sample. In 2018, the e-cigarette item was an undifferentiated umbrella, so a positive response alongside HTP confirms HTP use but not liquid-EC use; that cell is therefore reported as HTP with unconfirmed liquid-EC status, and confirmed dual use is not definable for 2018 (N/A). The 2019 e-cigarette item was liquid specific, so its dual-use cell is genuine. In contrast to 2019, a share of the 2018 unconfirmed cells are likely exclusive-HTP umbrella responders. Panel C: Among former-smoker HTP users in 2019, occasional users report a median of 10 days of use per month (IQR 3 to 10) within the 10-to-29-day band of the exposure question; among all adult current HTP users, 37% are occasional and are therefore counted as nonusers under a daily use exposure definition. No HTP frequency item existed in 2018; thus, daily use status is not definable for that wave. Percentages may not sum to 100 owing to rounding. Interquartile ranges for 2018 (years) are 0.25 to 1.50 (HTP/EC, all) and 5.00 to 20.00 (no-product, all); within the short-term stratum, 0.25 to 1.17 and 0.62 to 3.00; within the long-term stratum, 6.25 to 8.75 (n = 2) and 10.00 to 24.00.

4. Exposure definition and time-varying analysis

The exposure in the primary analysis is fixed at the 2018 screening. A participant who was a dual user at baseline and switched completely thereafter therefore remained in the current-smoker comparator for the whole follow-up period. Table S1 shows that the dual-user to switcher balance in Korea moved sharply in exactly that direction across the first year of follow-up, from 5.2:1 in 2018 to 2.1:1 in 2019; thus, this

misclassification is not hypothetical and runs in a single direction: the comparator progressively accumulates the exposure under test.

Time-varying exposure analysis

Kim et al. address this with time-varying models in which smoking status and e-cigarette use are treated as time-dependent covariates updated at the follow-up screening date. The analysis is restricted to the subpopulation rescreened from 2019 to 2020 (n = 3,465,275 of 4,524,895, or 76.6%), and the authors describe this as an exploratory subpopulation. Table S2 presents every estimate from that analysis beside its fixed-exposure counterpart, on the common contrast used in the correspondence: e-cigarette-using ex-smokers against ex-smokers not using a product, fully adjusted (model 2). All six estimates are lower under time-varying exposure than under fixed exposure. The direction of that shift is what misclassification of the comparator would produce, but the magnitude cannot be attributed to reclassification alone because the rescreened subpopulation is smaller and the event counts decrease correspondingly. Supplementary Tables 7 and 8 report only event counts and hazard ratios: no group sizes, no person-years, and no count of participants who changed exposure category. Without those, reclassification and reduced power cannot be separated, and we therefore report the pattern without attributing it to a single cause.

Table S2. Fixed-exposure and time-varying estimates compared e-cigarette-using ex-smokers vs. ex-smokers who did not use a product (model 2)

Outcome	Fixed: events	Fixed: aHR (95% CI)	Time-varying: events	Time-varying: aHR (95% CI)
Overall cohort				
Lung cancer incidence	71	1.56 (1.24-1.97)	31	1.38 (0.98-1.98)
Lung cancer-specific death	19	2.00 (1.28-3.15)	3	0.77 (0.25-2.39)
All-cause mortality	170	1.22 (1.05-1.41)	75	1.18 (0.94-1.48)
High-risk subgroup (50-80 yr, >=20 pack-years)				
Lung cancer incidence	49	1.91 (1.44-2.53)	14	1.40 (0.83-2.37)
Lung cancer-specific death	14	1.92 (1.13-3.24)	3	1.16 (0.37-3.61)
All-cause mortality	62	0.97 (0.76-1.25)	18	0.79 (0.50-1.25)

Fixed-exposure estimates from Kim et al.*, Extended Data Tables 1 and 3; time-varying estimates from Supplementary Tables 7 and 8. All are model 2 (adjusted for age, sex, pack-years, body mass index, household income, alcohol consumption, physical activity, Charlson comorbidity index, COPD and idiopathic pulmonary fibrosis), with ex-smokers not using a product as the reference. Note that the reference group key printed beneath Extended Data Tables 1 and 3 and Supplementary Tables 7 and 8 describes short-term and long-term cessation strata, which those four tables do not contain; the reference groups above are read from the rows marked as references in each table.*

What the deposited analysis code specifies

Kim et al. deposited the analysis code at https://github.com/SNUBH-bigdata/ECig_SmokingQuitters. It comprises two files. The R file is a plotting script that redraws Figure 3 from hazard ratios entered as literal values and performs no analysis. The SAS file defines six macros and invokes none of them; one macro it calls, %calc_incidence, is not defined in the file. No cohort derivation, no exposure construction, or

episode-splitting code for the time-varying dataset are included; thus, the exposure definitions and the construction of the time-varying person-time cannot be verified from the deposit.

Three specification details are nonetheless legible. First, the covariate list is identical in every model: age, sex, pack-years, body mass index, household income, alcohol consumption, physical activity, Charlson comorbidity index, COPD and idiopathic pulmonary fibrosis. Cessation duration is not a covariate in any model. It enters the analysis only as a level of the exposure classification in the five- and six-group analyses, and it is absent from the three-group classification used in every time-varying table. Second, age, pack-years and body mass index are not included in the CLASS statement; thus, each is entered as a single untransformed linear term on a time-on-study rather than an age time scale. Third, the three-group analyses are run with reference levels 1, 2 and 3 of the grouping variable and the five-group analyses with levels 1, 3 and 5. The reference group key printed beneath Supplementary Tables 7, 8, 17 and 18 describes short-term and long-term cessation strata, which the three-group variable does not contain; the estimates in those four tables are therefore against all product-free ex-smokers pooled.

What pooling does to the reference group

Because the time-varying analyses pool the cessation strata, the hazard of their reference group is set by its long-term majority. This information can be read directly from the article. Supplementary Tables 10 and 11 report the same cohort under the same any-use exposure definition, pooled and stratified, respectively, and their event counts are exactly reconciled; thus, pooling is the only difference between them. Among product-free ex-smokers, the short-term stratum contributes 6,971 lung cancer events at an adjusted hazard of 0.66 relative to current smokers, and the long-term stratum contributes 11,399 events at 0.51; the pooled value is 0.56, which is the event-weighted blend of the two (0.562). The exposed arm blends in the opposite direction, with 115 short-term events at 0.96 and 37 long-term events at 0.63, pooling to 0.85. The pooled contrast, 0.85 divided by 0.56, reproduces the reported 1.52 and is therefore a comparison between an exposed group that is 76% short-term by events and a reference group that is 62% long-term. Updating exposure status over follow-up does not alter that composition, and no covariate in the model represents it.

5. Cessation duration: the dominant confound

The two groups differ in how long ago they quit, and the difference does not go away when we account for it. Panel A shows the overall contrast (median 0.7 vs *12 years in 2018), Panel B shows that it holds within each of the two strata used by Kim* et al.*, and Panel C shows that it survives exact age matching. The overall contrast and the long-term stratum contrast are reproduced in every KNHANES wave from 2018 to 2023, and the age-matched residual ranges from 9 to 11 years across those waves; the two waves closest to the Kim* et al. *baseline are shown here.*

The same asymmetry appears in the study's own person-time, which is why the model adjusts the exposed cell using participants with whom the exposed cells barely overlap. Among HTP/EC-exposed participants, 27,046 of 136,459 person-years (19.8%) fall in the long-term cessation stratum; among quitters using no product, 7.56 of 11.64 million (64.9%) do. Within that stratum, the exposed contributes one person-year for every 279 in the reference stratum and one lung cancer event for every 714 (Kim et al., Table 2). The two groups therefore occupy opposite ends of the cessation–duration axis, and the covariate structure adjusting the 16 exposed events is drawn from a distribution that those events do not share.

This is the adjustment the correspondence describes. In the long-term stratum, the crude lung cancer incidence among HTP/EC switchers is 5.9 per 10,000 person-years, whereas it is 15.1 among no-product quitters (Kim et al., Table 2); thus, the unadjusted rate is 61% lower in the exposed group. Full adjustment (model 2) converts this into an adjusted hazard ratio of 1.74 (95% CI 1.07 to 2.84), a reversal of direction and a 4.5-fold change from the crude estimate. The exposed cells contribute 16 lung cancer events, compared with approximately 11,420 events in the reference. Sixteen events against ten covariates is approximately 1.6 events per covariate, well below the ten events per covariate conventionally treated as a minimum for stable Cox estimation; an adjustment of this size therefore rests on a cell too sparse to support it, drawn from a covariate distribution that the exposed does not share.

Table S3. Cessation duration among former smokers by product use group, KNHANES 2018 and 2019

Metric	2018	2019
A. Cessation duration, all former smokers (years)		
**HTP/EC group: median**	**0.7**	**2.0**
HTP/EC group: mean	1.2	2.0
HTP/EC group: n	43	101
**No-product group: median**	**12.0**	**12.0**
No-product group: mean	14.5	14.7
No-product group: n	1,305	1,326
B. Within-stratum cessation duration, median (years); n in parentheses		
**Short-term (<5 yr): HTP/EC**	**0.6 (41)**	**1.5 (92)**
**Short-term (<5 yr): no-product**	**2.0 (311)**	**2.0 (312)**
**Long-term (>=5 yr): HTP/EC**	**7.5 (2)**	**6.0 (9)**
**Long-term (>=5 yr): no-product**	**16.0 (994)**	**16.0 (1,014)**
C. Residual after exact age-matching (+/- 2 yr)		
HTP/EC: median age (yr)	39	40
Age-matched no-product: median cessation (yr)	10.0	11.0
**Residual cessation gap (yr)**	**9.3**	**9.0**
Maximum cessation among HTP/EC switchers (yr)	10.0	9.0
No-product quitters exceeding that maximum, %	53.3%	60.9%

HTP/EC group = former smokers currently using HTP or liquid EC; no-product group = former smokers using neither. The panel B stratum boundary is 5 years (short-term less than 5). Panel C: the no-product group is restricted to former smokers for whom at least one HTP/EC user falls within 2 years of age (n = 941 of 1,305 in 2018); the residual is the median cessation of that matched pool minus the median among all HTP/EC users. In 2018, the longest-abstinent HTP/EC switcher had quit 10.0 years earlier; in 2008, the year the authors gave for the arrival of e-cigarettes in Korea, 696 of 1,305 no-product quitters (53.3%), and 696 of 994 within the long-term stratum (70.0%), had quit longer ago than any switcher in the sample. The 2018 long-term HTP/EC median rests on two respondents and is reported for completeness only; it should not be read as a stable estimate. The long-term contrast is more securely read from 2019, when the

HTP/EC long-term cell contains nine respondents and the same ordering holds (6.0 vs *16.0 years), and it reproduces in every wave through 2023.*

Cessation duration of high-risk long-term quitters

Kim et al. reported no cessation duration for any group, so the duration of their high-risk long-term quitters, the comparator carrying the strongest subgroup claims, cannot be read from the article. It can be estimated by mapping that group onto KNHANES respondents matched on the covariates the article does report. Restricting KNHANES 2018 to no-product former smokers aged 56 to 71 (the mean plus or minus one standard deviation of Kim et al.'s long-term high-risk quitters; Extended Data Table 2), with at least 20 pack-years, who had quit at least 5 years before baseline (the article's long-term definition), yields a group matched on age and smoking intensity (Table S4).

The covariate match is close: the mean age is 64.0 years, whereas it is 63.6 years, and the mean number of pack-years is 33.8 and 30.4, respectively. The median cessation duration was 15.0 years (IQR 10.0 to 20.0), and 77% had quit at least 10 years before baseline. Because e-cigarettes reached Korea only in 2008, no switcher at the 2018 baseline could have been abstinent for more than approximately 10 years, and for the 79% using HTP, available from June 2017, the applicable ceiling is approximately one year. On this mapping, approximately three-quarters of the high-risk comparison group had been abstinent longer than the longest-possible switcher. This is an inference from an external survey rather than a measurement of the NHIS cohort; the two are national and contemporaneous but not identical, pack-years are reconstructed from self-reported duration and daily amount, and the matched cell contains 173 respondents.

Table S4. Cessation duration of KNHANES respondents matched to Kim et al.'s high-risk long-term quitters

Metric	KNHANES analog	Kim et al. group
Mean age (yr)	64.0	63.6
Mean pack-years	33.8	30.4
Median cessation (yr)	15.0 (IQR 10.0-20.0)	not reported
Quit >=10 years	77%	—
Quit >=15 years	53%	—
Quit >=20 years	31%	—
n	173	537 (long-term, e-cig)

KNHANES analog: no-product former smokers aged 56 to 71 years, at least 20 pack-years, who quit at least 5 years; KNHANES 2018 (n = 173). Kim et al. group: high-risk (50 to 80, at least 20 pack-years) long-term quitters using e-cigarettes, Extended Data Table 2 (n = 537); the article reports mean age and pack-years but no cessation duration. Cessation figures are estimated from the matched KNHANES respondents and are not measured in the NHIS cohort.

6. Age adjustment acting on sparse events: an empirical precedent

The concern that age adjustment can amplify rather than correct an estimate built on a handful of events is not hypothetical. Plurphanswat, Selya and Rodu reanalyzed the National Health Interview Survey (2014 to

2021) data underlying a widely cited report associating e-cigarette use with myocardial infarction (Plurphanswat N, Selya A, Rodu B. Questionable effects of electronic cigarette use on cardiovascular diseases from the National Health Interview Survey (NHIS, 2014-2021). Cureus. 2024;16(3):e57119. doi:10.7759/cureus.57119). Among never smokers who currently used e-cigarettes, 12 myocardial infarction events were observed. The crude odds ratio was 0.42 (95% CI 0.24–0.75); adjustment for every covariate except age left it essentially unchanged at 0.80 (0.45–1.43); adding age alone reversed it to 2.48 (1.35–4.55). The reversal was therefore produced by age acting on 12 exposed events whose ages sat in the upper tail of an otherwise young exposed group.

The structural parallel to that of Kim et al. is close: a protective crude estimate, an exposed cell of comparable size (16 lung cancer events in the long-term stratum), an exposed group approximately a decade younger than its reference, and an adjustment in which age performs nearly all of the work (Model 1 versus Model 2 differ by less than 2%). Whether the same amplification occurs here cannot be determined from the published tables because the age distribution of cases within each exposure group is not reported. We therefore note the possibility rather than assert it and identify the reporting that would resolve it: case age distributions by exposure group or an analysis stratified on age within the long-term stratum.

7. The cessation-duration gap holds even in a product-use population restricted to users affirmatively indicating that they used "electronic cigarettes" (broad term) 전자담배

The product composition of the NHIS-exposed group cannot be measured and is only bounded: on the two natural readings of a single e-cigarette question, HTP users make up between 50% and 79% of it (§2-3). Section 5 reports the cessation-duration contrast for the full product-using group, in which HTP users constitute the majority. This section asks whether that contrast depends on them. It does not. Under the narrowest reading, in which every exposed respondent affirmed the e-cigarette item, half of them also affirmed using an HTP. The median exposed respondent had quit six and a half months before the survey against twelve years in the comparator, and every quantity in Table S3 is preserved or strengthened.

Definition of the restricted group. Of the 43 product-using former smokers in KNHANES 2018, 18 answered the umbrella e-cigarette item (BS12_2 = 1); 9 of those 18 also reported HTP use on the separate checklist, and 9 reported liquid EC alone. The remaining 25 were HTP-only, identified by the checklist but not by the e-cigarette item. Restricting the exposed group to the 18 reproduces the narrow reading, under which every exposed respondent is an e-cigarette user by their own identification.

Results. The restricted definitions are compared against the full group in Table S5. The overall separation is essentially unchanged (D = 0.73 against 0.76; in both, the exposed respondent had quit more recently in 92% of cross-group comparisons). Within the short-term stratum, the separation is slightly wider (D = 0.37 against 0.34), and exact age matching leaves a residual gap of 9.5 years against 9.3 ($P < 0.001$ in both). The ceiling tightens: The longest-abstinent e-cigarette-item responders had quit 5.0 years before the survey, and 926 of 995 long-term comparators (93.1%) had quit longer ago than any exposed respondent did, compared with 70.0% in the full group. Within the 18 years, the 9 reporting liquid EC alone had a median cessation of 1.0 years, and the 9 also reporting HTP 0.3 years; both rest on nine respondents and are reported for completeness rather than as stable estimates.

What the restriction costs is precision, not effect size. Every measure of the size of the separation is preserved or slightly larger: D moves from 0.76 to 0.73 overall and from 0.34 to 0.37 within the short-term stratum, and the proportion of cross-group comparisons in which the exposed respondent had quit more

recently is unchanged at 92% and 73%, respectively; the age-matched residual widens from 9.3 to 9.5 years, and the age-by-group interaction coefficient is unchanged at -0.36. What moves are the P values, and they move with the number of exposed respondents rather than with the effect. In the short-term stratum, the number decreases from 41 to 17, and the Mann–Whitney P increases from less than 0.001 to 0.002 for an identical 73%. For the interaction, the age variance available within the exposed group decreases by approximately half (sum of squared deviations 4,108 to 2,154), the standard error increases from 0.17 to 0.23, and P increases from 0.03 to 0.12, while the estimate remains at -0.36. The interaction test therefore indicates what 18 respondents can resolve, not a smaller difference in slope. We do not lean on it in either analysis: the age-adjustment argument rests on the pattern visible in Figure 1C and reproduced in Figure S3C, in which cessation duration rises with age among no-product quitters but is recent at every age among product users. One quantity is genuinely lost rather than made imprecise: the exposed long-term stratum falls from two respondents to one, so the within-stratum comparison rests on the short-term stratum alone, and the long-term ordering is read from later waves, as in §5.

Table S5. Cessation duration under the full and restricted exposure definitions, KNHANES 2018

Metric	Full product-using group (Table S3)	Restricted to e-cigarette-item responders	Strict variant
Exposed group, n	43	18	18
Comparator group, n	1,305	1,330	1,305
A. Cessation duration, all former smokers (years)			
Exposed: median	0.7	0.5	0.5
Exposed: mean	1.2	1.1	1.1
Comparator: median	12.0	12.0	12.0
Comparator: mean	14.5	14.3	14.5
Kolmogorov–Smirnov D	0.76	0.73	0.75
Cross-group pairs in which the switcher quit more recently	92%	92%	93%
B. Within the short-term (<5 yr) stratum			
Exposed: median (n)	0.6 (41)	0.5 (17)	0.5 (17)
Comparator: median (n)	2.0 (311)	1.5 (335)	2.0 (311)
Kolmogorov–Smirnov D	0.34	0.37	0.38
Cross-group pairs	73%	73%	74%

Exposed respondents in the long-term (>=5 yr) stratum, n	2	1	1
C. Age adjustment			
Residual gap after exact age-matching (+/- 2 yr), years	9.3	9.5	9.5
Age-matched cross-group pairs	91%	90%	91%
Theil-Sen slope, exposed (yr per yr of age)	-0.01	0.00	0.00
Theil-Sen slope, comparator (yr per yr of age)	+0.30	+0.30	+0.30
Age x group interaction coefficient (SE; P)	-0.36 (0.17; 0.03)	-0.36 (0.23; 0.12)	-0.35 (0.23; 0.13)
D. Ceiling on exposed cessation duration			
Longest cessation among exposed respondents, years	10.0	5.0	5.0
Long-term comparators who had quit longer ago than any exposed respondent	70.0%	93.1%	93.1%

Full product-using group: former smokers reporting current use of liquid EC or HTP (Table S3). Restricted: former smokers answering the umbrella e-cigarette item, with the 25 HTP-only switchers reassigned to the comparator. Strict variant: the same 18 exposed respondents with the 25 HTP-only switchers excluded from both groups. All comparisons are two-sided and unweighted. The Mann–Whitney P values are less than 0.001 in every cell except for the short-term stratum under the restricted definition, where P = 0.002. The Kolmogorov–Smirnov P values in the short-term stratum are 0.019 (restricted) and 0.012 (strict), reflecting the exact test results for 17 exposed observations; in Panel A, all three values are less than 0.001. Interaction coefficients are the difference in ordinary-least-squares slopes with a pooled-variance t test; the Theil–Sen slopes are the robust estimates shown in the figures.

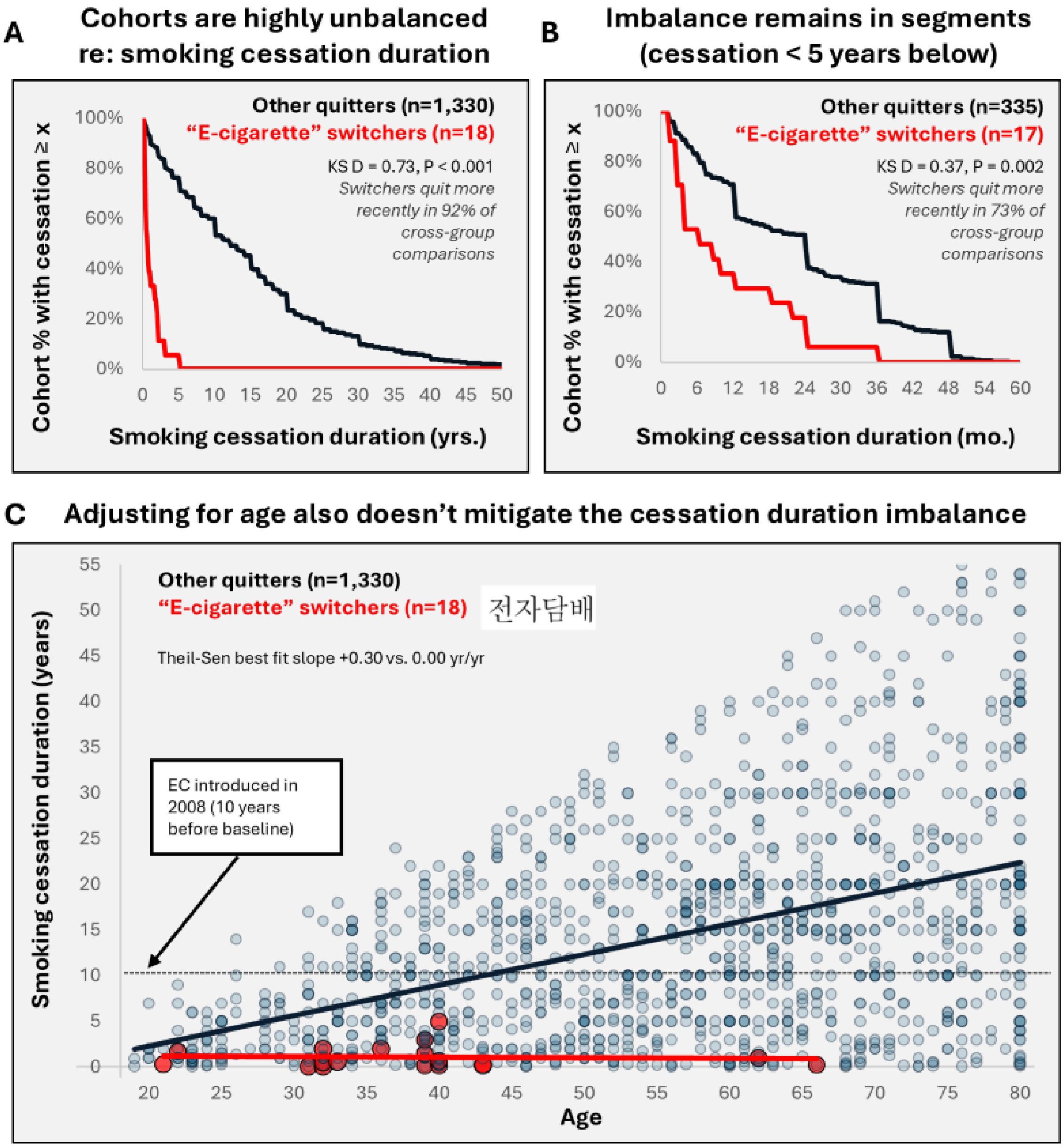

Figure S3. Smoking cessation duration under the restricted exposure definition, KNHANES 2018. *E-cigarette-item responders (red; n = 18)* vs *all other former smokers (blue; n = 1,330), (A) Complementary cumulative distribution across years, all former smokers (Kolmogorov–Smirnov D = 0.73; the exposed respondent had quit more recently in 92% of cross-group comparisons; P < 0.001). (B) The same in months within the short-term (<5-year) stratum (D = 0.37; 73%; Mann–Whitney P = 0.002). (C) Age* vs *cessation duration with per-group Theil–Sen fits; the dashed line marks cessation beginning in 2008, when e-cigarettes first went on sale in Korea.*
